# Immersion Pulmonary Edema in Female Triathletes

**DOI:** 10.1155/2011/261404

**Published:** 2011-06-01

**Authors:** Eric A. Carter, Michael S. Koehle

**Affiliations:** ^1^School of Human Kinetics, University of British Columbia, 210-6081 University Boulevard, Vancouver, BC, Canada; ^2^Division of Sports Medicine, University of British Columbia, Vancouver, BC, Canada

## Abstract

Pulmonary edema has been reported in SCUBA divers, apnea divers, and long-distance swimmers however, no instances of pulmonary edema in triathletes exist in the scientific literature. Pulmonary edema may cause seizures and loss of consciousness which in a water environment may become life threatening. This paper describes pulmonary edema in three female triathletes. Signs and symptoms including cough, fatigue, dyspnea, haemoptysis, and rales may occur within minutes of immersion. Contributing factors include hemodynamic changes due to water immersion, cold exposure, and exertion which elevate cardiac output, causing pulmonary capillary stress failure, resulting in extravasation of fluid into the airspace of the lung. Previous history is a major risk factor. Treatment involves immediate removal from immersion and in more serious cases, hospitalization, and oxygen administration. Immersion pulmonary edema is a critical environmental illness of which triathletes, race organizers, and medical staff, should be made aware.

## 1. Introduction

Immersion Pulmonary Edema (IPE) is a rare but potentially life-threatening condition first reported in otherwise healthy SCUBA divers in 1984 [1]. Recently, an increasing number of cases have been published in apnea divers and long-distance military swimmers [2–4].

In a review of IPE cases, dyspnea, cough, and haemoptysis were the most commonly reported symptoms while loss of consciousness and cardiopulmonary arrest occurred infrequently [4]. Onset of symptoms is rapid and exacerbated by exertion [2].

Anecdotal reports of symptoms similar to IPE have recently begun to emerge within the Triathlon community. A recent survey distributed to the USA Triathlon Organization (USAT) received 1400 responses and estimated the population prevalence of IPE at 1.4% [5]. This is similar to the prevalence of 1.8% reported in military combat swimmers [6]. Miller et al. found that history of hypertension, fish-oil supplements, wetsuit use, and long-distance swims were statistically significant risk factors for IPE [5]. 

## 2. Case Reports

### 2.1. Case 1

In May 2009, a 58-year-old Caucasian woman experienced severe shortness of breath and haemoptysis while training for a triathlon in cold fresh water (15°C). The patient entered the water from a floating dock and was immediately submerged but denies water aspiration. Dyspnea developed upon surfacing and continued while swimming 250 meters further. At this point, breathing was difficult and a cough developed. After a total of 30 minutes in the water, her cough became productive with pink frothy sputum. Extreme fatigue and coughing necessitated 25 to 30 minutes of slow swimming to return to shore as well as assistance exiting the water. Immediately upon exiting the water, symptoms included shortness of breath, cough, haemoptysis, chest tightness, crackles and rales, extreme fatigue, and irregular breathing. Cough and haemoptysis persisted for five hours until presentation at the emergency room. On admission, her pulse was 81 bpm, her blood pressure was 158/85 mmHg and her oxygen saturation (S_P_O_2_) was 94%. A chest radiograph (CR) taken on admission showed a patchy opacity within the left upper lobe anterior segment. Follow-up CR taken one day after admission showed no significant abnormality. Lab work was obtained immediately on admission and a followup on parameters outside the normal range from each report are listed in Table 1.

The patient suffered three more episodes, all in cold (<22°C) fresh water. During the second and third episodes, which were training swims, symptoms began within 15 min. On shore, coughing and haemoptysis continued. During the fourth episode, a triathlon race, symptoms began within five min and the patient was forced to exit the water via a rescue boat. Medical staff reported elevated blood pressure and offered oxygen which was declined. After each episode, dyspnea, cough, haemoptysis, and fatigue resolved within 2-3 hours and no hospitalization was required. 

Patient's medical history includes no major cardiopulmonary disease other than a possible diagnosis of mild asthma as a result of temporary symptoms following a 2005 viral infection. She stopped smoking in 1995 and has a family history of premature cardiovascular disease. Echocardiogram was normal with normal pulmonary artery pressure. Electrocardiogram and maximal exercise test were normal; she had a blood pressure of 116/56 and is of a normal weight. Between these episodes, she has successfully completed two open water triathlons and swims in Lake Ontario three times per week. Prior to the first episode, she reported consuming 0.5 L water and one large cup of coffee. Prior to subsequent episodes, she reported salbutamol (Ventolin, GlaxoSmithKline, London, UK) use and minimal fluid intake. All episodes occurred while wearing a wetsuit.

### 2.2. Case 2

**Figure 1:**
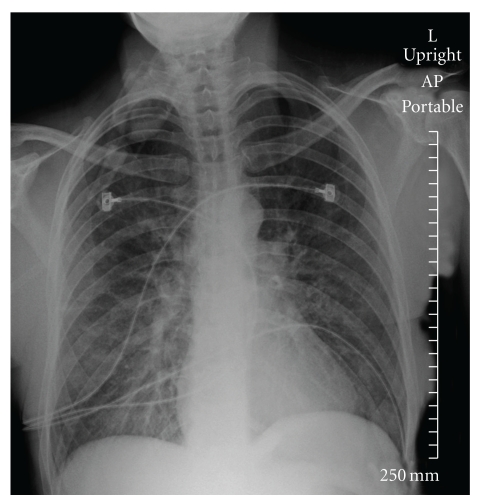
Case 2 chest X-ray at time 1744. Abnormal chest X-ray taken on admission to the emergency room. shows bilateral airspace with ground glass predominately on the lower right side. The image shows no Kerley B lines, vascular redistribution, or pleural effusions. Heart size appears normal.

**Figure 2:**
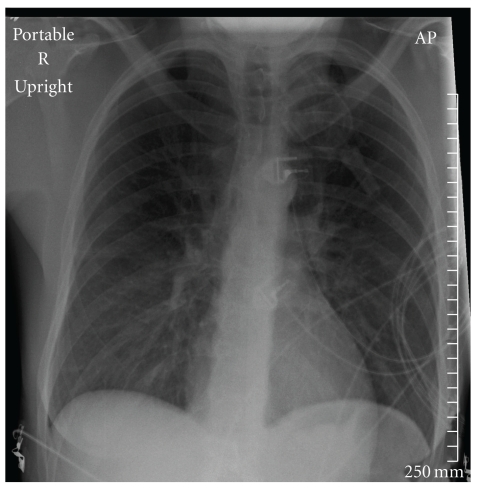
Case 2 chest X-ray at time 1944. Chest X-ray taken two hours after admission shows slight ground glass abnormalities despite significant improvement.

In June 2010, a 45-year-old Caucasian woman developed severe shortness of breath during a training swim, one day after completing a half-Ironman distance triathlon. After swimming 30 minutes in cold (18°C) fresh water, the patient experienced chest tightness. Ten minutes later, severe dyspnea prevented her from continuing the swim and she was assisted to shore. Immediately upon exiting the water, symptoms included shortness of breath, cough, chest tightness, rales, extreme fatigue, irregular breathing, and cyanosis. The patient had no recollection of water aspiration. On admission to the local hospital, her S_P_O_2_ was 85%. Thirty minutes following admission, CR showed bilateral ground glass predominately on the lower right side (Figure 1). Follow-up CR two hours later showed significant improvement (Figure 2). 

This patient had a history of asthma and environmental allergies. She consumed approximately one liter of water prior to the swim as well as two puffs of budesonide/formoterol (Symbicort, AstraZeneca, London, UK). She was wearing a wetsuit and complained of it feeling overly tight.

### 2.3. Case 3

In June 2007, a 43-year-old Caucasian woman experienced shortness dyspnea, chest tightness, and cough after swimming 750 meters of a half-Ironman distance triathlon. Symptoms began after 10 min of swimming in cold (19°C) fresh water. The patient completed the 1,930 meter swim as well as the 90 km bicycle ride but dropped out during the run. Paramedics treated her for a presumed diagnosis of hypothermia and low S_P_O_2_ with supplemental oxygen.

In July 2007, she experienced similar symptoms after 11 min of swimming in cold (22°C) fresh water while competing in an Ironman distance triathlon. Upon exiting the water, the patient was seen in the race medical tent where her symptoms included chest tightness, dyspnea, haemoptysis, and rales. Medical staff reported her S_P_O_2_ as 76% then administered furosemide (Lasix, Sanofi-Aventis, Paris, France) and supplemental oxygen. On admission to a local hospital, CR confirmed pulmonary edema.

Additional episodes occurred in September 2008 and June 2010 after finishing Ironman distance triathlons in Canada and France. Both episodes occurred in cold (<23°C) fresh water. 

The patient has no outstanding medical history other than environmental allergies. A maximal exercise stress echocardiogram under the supervision of her cardiologist proved inconclusive. With a blood pressure of 140–160/82–96, she was diagnosed with mild hypertension and prescribed candesartan (Atacand, AstraZeneca, London, UK) (16 mg). During each episode she wore a wetsuit. She has completed multiple half and full Ironman distance triathlons before and during these episodes.

## 3. Discussion

Previous anecdotal reports of triathletes, as well as military swimmers and divers, experiencing episodes of IPE exist in the scientific literature [2, 5]. Prior to this paper, no cases in triathletes have been confirmed with imaging. This is the first documented case series of any female triathletes who have received a diagnosis of IPE confirmed by CR. 

IPE is believed to be caused by a combination of water immersion, cold, and exertion which act to increase cardiac output (Q), elevating pulmonary artery pressure (P_PA_). When P_PA_ is elevated beyond a certain point, capillary stress failure is likely to occur. Fluid is then forced from the capillary to the interstitial space and at higher pressures, into the alveolar space of the lung. Increased hydrostatic pressure due to immersion causes a redistribution of blood from the periphery to the core associated with an increase in intrathoracic blood volume of 700 mL. This can result in P_PA_ increasing up to 12 mmHg [7]. Cold causes peripheral vasoconstriction and blood is redistributed from peripheral to thoracic vessels resulting in increased preload and afterload [8]. Cardiac output was found to increase significantly during cold water immersion [6]. Elevated Q during exercise is the result of increases in oxygen consumption, ventilation, heart rate, and stroke volume [9].

All three cases consist of women between age 43 and 59 participating in triathlon training swims or races (Table 2). This supports evidence by Miller et al. suggesting that women and those over the age of 40 are likely to have increased odds of experiencing pulmonary edema [5]. Similarly, Koehle et al. found that older SCUBA divers are more likely to develop IPE [2]. Each woman experienced similar symptoms including dyspnea, cough, haemoptysis, and extreme fatigue. During most episodes, patients were able to return to shore by swimming slowly however two of the women each describe one episode where they feared for their life but were assisted to shore by lifeguards or fellow swimmers. 

IPE incidents may be underreported in triathletes. Triathletes are often highly intrinsically motivated and may attempt to complete a race despite adverse events; if an athlete suffers a minor case of IPE, it is likely to resolve quickly upon exiting the water and the athlete may only think they had a “bad day”, declining to seek medical attention. Many athletes, race organizers, and even medical staff are unaware of IPE which results in an incorrect diagnosis such as water aspiration. It has been suggested that wearing a wetsuit will increase the odds ratio of developing pulmonary edema [5]. The third patient in this series reported an overly tight wetsuit adding to sensations of dyspnea. An overly tight wetsuit may increase the effects of hydrostatic pressure exerted on the body and contribute to elevated pulmonary artery pressure and a central redistribution of blood volume. 

The most important implications of this case series are those for race organizers and staff. Recognition and quick removal from immersion are crucial for patients with IPE. Symptoms can begin quickly (<10 min), even in relatively warm water. Race organizers should ensure that medical staff are educated on the field diagnosis and management of IPE. Furthermore, organizers must equip their staff with equipment and training to immediately remove dyspneic, coughing swimmers from the water and to administer supplemental oxygen.

For athletes, it is important to be aware of IPE risk factors and how to manage them. They must also be able to recognize the symptoms of IPE before becoming incapacitated. Currently, no treatment has been proven to prevent the onset of IPE. Anecdotally, Nifedipine has been used as a prophylactic with some success in SCUBA divers. The long-term implications of repeated IPE incidents is unknown. All three triathletes detailed in this report have continued to train and compete successfully, however further study is needed before this can be recommended. Because the possibility of reoccurrence is high, the risk of drowning still exists.

## 4. Conclusion

IPE is a serious environmental illness, likely triggered in triathletes by cold water immersion and exertion and may be exacerbated by other factors including gender, age, wetsuit tightness, and hydration status. Many patients experience multiple episodes of IPE, making previous history a major predisposing factor. IPE occurs in otherwise healthy males and females immersed in fresh or salt water. IPE generally occurs in colder water but has been described in warm water as well. Symptoms generally resolve quickly when patients are removed from water.

## Figures and Tables

**Table 1:** Case 1 laboratory results, May 2009.

May 30 2009	May 31 2009
Complete Blood Count	Complete blood count
(i) Leukocytes: above normal (19.2 × 10 g/L) (ii) Mean Cell Volume: below normal (80.7 fL) (iii) Mean cell HB: below normal (26.3 pg)	(i) Leukocytes: above normal (11.9 × 10 g/L) (ii) Hemoglobin: below normal (111 g/L) (iii) Hematocrit: below normal (0.333 L/L) (iv) Mean Cell Volume: below normal (79.4 fL) (v) Mean cell HB: below normal (26.5 pg)

Differential	Differential
(i) Neutrophil: above normal (17.76 ×10 g/L) (ii) Lymphocyte: below normal (0.92 × 10 g/L)	(i) Neutrophil: above normal (9.8 ×10 g/L)

Blood Gas, Venous	Blood gas, venous
(i) pO_2_: below normal (30 mmHg) (ii) O_2_ Saturation: below normal (0.55)	(i)pH: above normal (7.42) (ii)pCO_2_: below normal (39 mmHg) (iii)pO_2_: above normal (79 mmHg) (iv) O_2_ Saturation: above normal (0.96)

**Table 2:** Characteristics of the case patients.

Variable	Case 1	Case 2	Case 3
Sex	Female	Female	Female
Age, yr	58	45	43
Height, cm	157	170	163
Weight, kg	56	57	57
Number of Episodes	4	1	4

## Abbreviations

CO: Cardiac output
CR: Chest Radiograph
IPE: Immersion pulmonary edema
P_PA_: Pulmonary artery pressure
S_P_O_2_: Saturation of peripheral oxygen
USAT: United States of America Triathlon.
